# Ordering from the bill instead of from the menu

**DOI:** 10.1002/ccd.29822

**Published:** 2021-06-17

**Authors:** Andrew M. Goldsweig, Eric Secemsky, Rishi Wadhera, David J. Cohen

**Affiliations:** 1 Division of Cardiovascular Medicine, University of Nebraska Medical Center, Omaha, Nebraska, USA; 2 Department of Internal Medicine, Smith Center for Outcomes Research in Cardiology, Beth Israel Deaconess Medical Center, Boston, Massachusetts, USA; 3 Division of Cardiovascular Medicine, Harvard Medical School, Boston, Massachusetts, USA; 4 Cardiovascular Research Foundation, New York, New York, USA; 5 Division of Cardiovascular Medicine, St. Francis Hospital, Roslyn, New York, USA

Outcomes research based upon administrative claims databases is a large and growing field within interventional cardiology. Claims databases are compiled routinely for billing and related health care administrative uses by government and commercial payers. These data may be repurposed to study procedure utilization and outcomes. This journal alone has published more than 90 studies using data from United States federal government administrative claims databases, 59 of them since 2015. However, administrative claims databases are not engineered for clinical research, and as a result, they can be difficult to use accurately.^1^

Numerous studies from multiple databases have reported declining rates of both coronary artery bypass grafting (CABG) and percutaneous coronary intervention (PCI): the Washington Cardiac Care Outcomes Program (CABG 1.7% and PCI 0.2% per year 2005–2017),^2^ the Massachusetts Department of Public Health (CABG 4.4% and PCI 3.7% per year 2003–2012),^3^ and the National Cardiovascular Data Registry (NCDR; PCI 3.0% per year 2010–2014)^4^ have all documented declining rates of coronary artery revascularization procedures. More recently, as published in this journal, Shah et al. used claims data from the Centers for Medicare and Medicaid Services (CMS) to examine national rates of CABG and PCI as well as physician reimbursement between 2010 and 2018 and reported that, over this time period, per capita rates of CABG and PCI decreased by 40.7% and 26.4%, respectively, equating to 5.1% and 3.3% per year.^5^ While this trend is qualitatively consistent with prior studies, the magnitude of these figures ranks among the highest annual decrease reported.

As such, it is important to consider the basis for these calculations. To determine the rate of revascularization per Medicare enrollee, this study counted the number of CABG and PCI procedures (“the numerator”) and the number of Medicare enrollees (“the denominator”). For the numerator, the number of CABG and PCI procedures was extracted from the Medicare Part B National Summary Data File, a representative sample of all Medicare fee-for-service patients. By using physician claims as the basis for calculating procedure volumes, this approach overcomes challenges posed by the transition of many elective PCI procedures to the outpatient setting, where they are not captured by many claims databases that focus specifically on inpatient services (such as the National Inpatient Sample and Nationwide Readmissions Database).^6^ For the denominator, the number of Medicare enrollees was extracted from Medicare Enrollment Reports, including both fee-for-service Medicare as well as Medicare Advantage.

Medicare Advantage or “Part C” is a group of managed care plans offered through Medicare but administered by commercial insurers. For many patients, these plans are attractive because they provide greater coverage (especially prescription drug coverage) and additional benefits (e.g., disease management coaching) compared with traditional fee-for-service Medicare. Because these plans are administered by third parties rather than CMS, their claims are generally excluded from most Medicare databases including Medicare Part B. Of note, between 2010 and 2018, Medicare Advantage enrollment increased from 11.1 million (24% of beneficiaries) to 20.4 million (34% of beneficiaries).^7^

Thus, in this study, the denominator continued to increase each year with the overall growth of Medicare, but the numerator was drawn from the shrinking cohort of Medicare fee-for-service patients. As a result, the calculations of CABG and PCI volumes per-enrollee overstated the true rate of decline of these procedures. Based on the Medicare fee-for-service population, we calculate the true rates of decline of CABG and PCI between 2010 and 2018 to be approximately 31.0% and 14.3%, respectively (3.9% and 1.8% per year). The calculations indicating that per capita physician payments for coronary revascularization decreased by 49.3% for CABG and 36.6% for PCI (5.5% and 4.1% per year, respectively) are similarly erroneous. Using the proper denominator, we calculate a decrease in Medicare expenditure per enrollee to be 41.0% and 45.7% for CABG and PCI, respectively (5.1% and 5.7% per year). Even these revised calculations are incorrect, however, as physician reimbursement for CABG in 2018 was at least ~$1800/procedure and up to ~$2400 using all arterial grafts—far greater than the ~$600/procedure reported in the paper. The reasons for this large discrepancy cannot be discerned from the methods provided but may relate to failure to capture multiple CPT billing codes associated with CABG operations (e.g., separate codes for vein harvesting, arterial grafts, venous grafts, etc.).

These issues highlight some of the challenges in the use of administrative claims data for outcomes research. For example, in order to capture the proper patient cohort, the correct set of claims codes must be identified from among more than 69,000 presently available codes. In addition, the coding system switched entirely from the International Classification of Diseases (ICD) 9 to ICD 10 on October 1, 2015. Most importantly, each database captures a different population and time period, which may not fit the proposed research precisely (Table 1).

By way of analogy, performing research based upon administrative claims data may be likened to a restaurant diner ordering from the bill instead of from the menu. Confusing like claims data, the bill lists only a small fraction of the available dishes, and the details are distorted by taxes and gratuities. The menu, on the other hand, is designed for ordering and provides a wide range of more accurate information (ingredients, portion size, etc.). Fortunately, “menu-like” databases can be designed for clinical research, capturing specific data of interest to researchers using standardized definitions. In cardiology, databases designed for clinical research are increasing in number, such as the 11 NCDR registries.^8^ Yet even “built for purpose” clinical databases are not ideal for every research question. Indeed, clinical databases and claims databases often contain complementary information, and recent efforts have focused on merging these two types of data to provide the advantages of both. Ultimately, it is the responsibility of the investigators to understand the specific characteristics of each dataset so that it can be applied correctly to the question under investigation. Otherwise, by ordering from the bill instead of from the menu, as when conducting research using administrative claims, what you get may not be what you wanted.

## Figures and Tables

**TABLE 1:** Details of selected administrative claims databases compiled by government and private payers in the United States

Database	Organization	Population and website
National Inpatient Sample (NIS)	US Department of Health and Human Services	• 20% stratified sample of all-payer discharges in 49 states • ~7 million discharges per year (representing ~35 million nationally • Only inpatient data; patients cannot be tracked across multiple admissions • www.hcup-us.ahrq.gov/nisoverview.jsp
Nationwide Readmissions Database (NRD)	US Department of Health and Human Services	• Stratified sample of all-payer discharges in 28 states • ~18 million discharges per year (representing ~35 million nationally) • Only inpatient data; patients can be tracked across multiple admissions but cannot be tracked between calendar years • Deaths only recorded if they occur during an admission • www.hcup-us.ahrq.gov/nrdoverview.jsp
Medicare Files	Center for Medicare and Medicaid Services	• Enrolled Americans ages ≥65 • >30 different demographic, inpatient, and outpatient files available • Can be costly to obtain and require a detailed proposal and data use agreement • https://www.cms.gov/Research-Statistics-Data-and-Systems/Research-Statistics-Data-and-Systems
Premier Healthcare Database	Premier Inc.	• All-payer discharges and outpatient encounters from participating hospitals • ~10 million discharges per year (representing ~25% of all US admissions) • ~93 million outpatient encounters per year • ~231 million unique patients • Patients can be tracked longitudinally but only at participating hospitals • Costly to access • Products.premierinc.com/applied-sciences
OptumLabs Data Warehouse	Optum, Mayo Clinic	• Commercial insurance and Medicare Advantage enrollees • Inpatient and outpatient data • ~200 million unique patients • Patients can be tracked longitudinally • Costly to access • Data analyses performed by central contractor rather than investigator • www.optumlabs.com
